# Evolution of universal review and disclosure of MRI reports to research participants

**DOI:** 10.1002/brb3.428

**Published:** 2016-02-08

**Authors:** Jody M. Shoemaker, Caitlin Cole, Linda E. Petree, Deborah L. Helitzer, Mark T. Holdsworth, John P. Gluck, John P. Phillips

**Affiliations:** ^1^ The Mind Research Network1101 Yale Blvd NEAlbuquerqueNew Mexico87122; ^2^ Department of Family and Community MedicineUniversity of New Mexico Health Science Center1 University of New MexicoAlbuquerqueNew Mexico87131; ^3^ Clinical and Translational Science CenterUniversity of New Mexico Health Science Center1 University of New MexicoAlbuquerqueNew Mexico87131; ^4^ College of PharmacyUniversity of New Mexico Health Science Center1 University of New MexicoAlbuquerqueNew Mexico87131; ^5^ Kennedy Institute of EthicsGeorgetown UniversityHealy Hall, 4th FloorWashingtonDistrict of Columbia20057; ^6^ Department of PsychologyThe University of New Mexico1 University of New MexicoAlbuquerqueNew Mexico87131; ^7^ Department of NeurologyUniversity of New Mexico Health Sciences Center1 University of New MexicoAlbuquerqueNew Mexico87131

**Keywords:** Incidental findings, magnetic resonance imaging, neuroimaging, research findings disclosure

## Abstract

**Background:**

Although incidental findings (IF) are commonly encountered in neuroimaging research, there is no consensus regarding what to do with them. Whether researchers are obligated to review scans for IF, or if such findings should be disclosed to research participants at all, is controversial. Objective data are required to inform reasonable research policy; unfortunately, such data are lacking in the published literature. This manuscript summarizes the development of a radiology review and disclosure system in place at a neuroimaging research institute and its impact on key stakeholders.

**Methods:**

The evolution of a universal radiology review system is described, from inception to its current status. Financial information is reviewed, and stakeholder impact is characterized through surveys and interviews.

**Results:**

Consistent with prior reports, 34% of research participants had an incidental finding identified, of which 2.5% required urgent medical attention. A total of 87% of research participants wanted their magnetic resonance imaging (MRI) results regardless of clinical significance and 91% considered getting an MRI report a benefit of study participation. A total of 63% of participants who were encouraged to see a doctor about their incidental finding actually followed up with a physician. Reasons provided for not following‐up included already knowing the finding existed (14%), not being able to afford seeing a physician (29%), or being reassured after speaking with the institute's Medical Director (43%). Of those participants who followed the recommendation to see a physician, nine (38%) required further diagnostic testing. No participants, including those who pursued further testing, regretted receiving their MRI report, although two participants expressed concern about the excessive personal cost. The current cost of the radiology review system is about $23 per scan.

**Conclusions:**

It is possible to provide universal radiology review of research scans through a system that is cost‐effective, minimizes investigator burden, and does not overwhelm local healthcare resources.

## Introduction

Improvements in magnetic resonance imaging (MRI) technology over the past decade have increased the ability of clinicians and investigators to identify individual differences in the brain structure. With these enhancements also comes the likelihood of identification of findings that may be unexpected and potentially clinically significant. In approximately 30% of individuals who undergo an MRI, incidental findings (IF) are identified – discoveries that are outside the purpose of the research study, most of which have little or unknown medical significance (Katzman et al. 1999; Vernooij et al. 2007; Royal and Peterson 2008; Illes and Borgelt 2009). What to do with this information is a matter of significant controversy. The decision regarding whether to review research MRI scans for IF is left to individual imaging centers, or in some cases, individual investigators. As a result, at least four distinct radiology review and disclosure models are currently in practice across the United States (Illes and Chin 2008; Lo 2010; Rangel 2010; Shoemaker et al. 2011; Booth et al. 2012; Anastasova et al. 2013; Thorogood et al. 2014). These include: (1) no review – MRI scans are not reviewed for potential clinical findings, no findings are returned to the participants and the scans are only utilized for the specific purpose of the research study; (2) select review – the MRI technologist or researcher conducting the scan can “flag” any scans with suspicious‐looking findings for further review by a radiologist or neurologist, and limited findings may be returned to participants; (3) full review – all research scans are reviewed by a radiologist and all findings are reported to participants; (4) and finally, some research centers, including the National Institute of Mental Health, conduct a state‐of‐the‐art clinical MRI scan, which is reviewed by a radiologist, and the results are provided to the participant.

The National Institute of Health (NIH), recognizing the need for greater consistency among research centers, in 2005 and then again in 2012, supported working groups whose members were asked to discuss IF in neuroimaging research (Illes et al. 2006; Underwood 2012). These groups made recommendations to help streamline existing guidance from NIH concerning: participant opt‐out options, inclusion of qualified professionals for scan review, budgetary allowances to cover the cost of review and disclosure, and language regarding potential IF in the consent forms. Similarly, the recent Presidential Commission for the Study of Bioethical Issues called for investigators to “Anticipate and Communicate” IF across clinical, research and direct‐to‐consumer contexts (Presidential Commission for the Study of Bioethical Issues. 2013). Several recommendations were particularly relevant to MRI research: plans for identifying and reporting IF should be part of the informed consent process and specialist referral should be provided if needed; however, researchers should not be obligated to look for IF, particularly if doing so would burden and undermine the research enterprise. Despite these recommendations, no consensus was reached by either group regarding whether all research scans should undergo a radiology review and how much, if any, information should be returned to participants.

Previous literature indicates that the vast majority of research participants express a preference to know what their MRI scans reveal regardless of the clinical significance (Kirschen et al. 2006; Illes et al. 2008; Wolf et al. 2008; Phillips et al. 2015). It has been suggested that providing participants with this information supports the ethical principles of individual autonomy and beneficence. However, some stakeholders, specifically investigators and research administrators, have raised concerns that conducting a formal radiology review of research MRI scans and giving participants their radiology reports would cause an excessive burden on research budgets, increase participant anxiety, and expand legal liability (Milstein 2008; Royal and Peterson 2008; Shaw et al. 2008; Deslauriers et al. 2010; Orme et al. 2010; Schmidt et al. 2013; Wardlaw and Jackson 2013; Mason 2014). Unfortunately, little empirical data exists to guide researchers and institutions to balance these competing priorities.

This article aims to address some of this knowledge gap by reporting on the development and outcomes of a system of universal radiology review at a single large neuroimaging facility. First, the development procedures, administrative details, financial analysis, and mechanisms of automation including remote neuroradiology review and Medical Director oversight are reported in detail. Second, the findings from a large stakeholder survey including participant interviews are provided to illuminate the personal and institutional impact of this universal review and disclosure system. The combined information from these elements, arising from our experience with over 15,000 research MRI scans suggests that, in practice, the burden of performing radiology reviews and providing reports to the participants is minimal.

## Methods

### Site characterization

As summarized in Shoemaker et al., our site is a nonclinically‐based, independent nonprofit, imaging research institute, which specializes in neuroimaging (MRI, MEG, EEG). MRI scans from 216 studies by 63 investigators using three scanners (Siemens 1.5 and 3 Tesla) have been reviewed for findings since 2004. The sequences obtained varied according to the study protocol and usually included at least one anatomic scan; complete clinical scans were not part of any study. As mandated by our local IRB, all research MRI scans that contain readable, structural sequences are reviewed by a radiologist for IF. The organization includes departments of Neuroinformatics, Information Technology (IT) and Imaging. A part‐time Medical Director (a board‐certified neurologist) is contracted through a local medical school, and two board‐certified radiologists review the MRI scans as independent contractors. The research areas typically focus on neurologic and psychiatric disorders with an emphasis on addictions, psychosis, psychopathy, neurodevelopment, and traumatic brain injury. The study participants include individuals with diagnosed illnesses as well as healthy control subjects.

### Radiology review process

The current institution‐wide process for evaluating the research MRI scans involves a review by a radiologist. The radiologist's summary, along with a binary referral recommendation, is returned to every participant scanned at our imaging center. Over the past decade, the annual number of radiology reviews completed increased from approximately 100 in the first year to a current average of approximately 1800 per year. In addition to the report provided to each participant, the study Principal Investigator (PI) and the Medical Director also receive e‐copies of each report generated. A summary of the various specific system enhancements and logistical details is provided below.

#### Radiologist

All MRI scans that contain structural images are reviewed unless a review had been completed for that participant within the previous 6 months (or previous 3 months for children 2 years of age or younger). These time points were determined by the Medical Director and radiologists from their clinical experience in estimating the minimal time required for a new radiographically evident lesion to develop in an asymptomatic individual. The radiologists review the current scans at least once a week. In addition, if a finding is identified by the MRI technologist as being of potential medical concern at the time of scanning, the radiologist and Medical Director are notified via an automatically generated e‐mail and an immediate review of the potential abnormality is performed. This process, which depends on the study, IF severity and the age of the subject, allows for targeted and timely IF reviews of scans. On rare occasions (<1/year), in order to rule out false‐positive findings, a short repeat MRI scan can be completed on‐site as part of the institution's IF review process to clarify the radiological findings.

#### Medical Director

Over time, the Medical Director's role has evolved to remain time efficient while still meeting system needs. Automated features, such as priority flags on suspicious scans and e‐mailed radiology reviews now notify the Medical Director immediately of any potentially serious findings. For the very rare *urgent* findings, the Medical Director telephones the participant (or parent/guardian) prior to the report arriving in the mail to discuss the finding and clarify any questions regarding the significance of the finding. Additionally, the Medical Director's name and contact information is provided on all radiology review reports so that research participants may call with questions. The role of the Medical Director can thus be conceptualized as augmenting the automated process to ensure that research participants have access to a qualified professional to clarify their radiology report and offer general information about the reported findings and next steps for clinical care.

An additional task of the Medical Director is to facilitate, if necessary, the transition from research participant to clinical patient when medically important findings are identified. Because most IF fall into predetermined referral categories, vetted through the Medical Director and local specialists ahead of time, the recommendation for clinical follow‐up in most cases has already been determined based on the type and severity of IF identified. The Medical Director is also able to assist research participants in getting the help they need, by obtaining specialist referrals or helping them navigate the local medical system. Thus, the automated system helps prioritize the Medical Director's time to those cases requiring personal involvement. This level of assistance fulfills the ethical requirements incumbent on clinical research, which is more fully explored in Phillips et al. 2015. Administrative staff documents all communication and correspondence between the Medical Director and research participants. Furthermore, the primary responsibilities of the Medical Director can be managed remotely, which facilitates multisite collaborations and services for sites that do not have a Medical Director or a radiology review process in place.

#### Neuroinformatics

Over the past few years, significant system enhancements have been implemented to the internally developed neuroinformatics software system COINS (COllaborative Informatics Neuroimaging Suite: www.coins.mrn.org) (Bockholt et al. 2010). The original radiology review feature in COINS was designed for on‐site review by a single radiologist. Using secure cloud‐based storage, Virtual Private Network, and team viewer software (www.teamviewer.com), the reviews are now completed remotely by multiple radiologists. The completed scans are synchronized with the cloud storage every hour, allowing for rapid review when necessary. Separate worklists are automatically generated for each radiologist based on the study, the priority of the finding, and the radiologist's expertise. Previously, all MRI sequences, including functional tasks and other data of little utility to the radiologist were uploaded for review. Currently, only structural sequences with clinical utility, as determined by the study PI and the Medical Director are uploaded and reviewed. The reduction in the data uploaded saves on transmission time and storage space and improves the radiologists' productivity. In addition, transferring only the required data to our radiologists, and never using subject identifiers, ensures research participant confidentiality.

#### Cover letter and reading templates

The information contained in the radiology review cover letter and the report has been modified since the disclosure process was initiated. From 2005 to 2012, a 5‐point scale adapted from Katzman was used to describe the type and urgency of any follow‐up of the findings that was required (Katzman et al. 1999). On the basis of feedback from investigators, radiologists, local physicians, and participants, the referral system was simplified in 2013, and the cover letter was modified. The overwhelmingly common theme from all groups was that the participants needed and wanted to know when they should see a doctor about the findings noted on their radiology review. To meet this need, the new template includes a radiologist summary of any findings and a referral recommendation that is either: “You do not need to see your doctor about this report” or “Please see your doctor about this report” (appendix 1 and 2).

A major step in our system's evolution was standardizing radiology review language and referral recommendations. Based on several years of experience, it was recognized that most findings identified fit into one of a number of potential diagnoses (appendix 3). This provided an opportunity to standardize IF management. Working with local specialists, current medical literature was reviewed and decisions were made about which findings in an asymptomatic individual require further medical evaluation. All common findings were categorized into one of the two recommendations (doctor referral or no doctor referral necessary) based on specific information such as size (e.g., pineal cyst greater than 1 cm), location (e.g., unilateral maxillary sinus opacification), and other important distinguishing features. The creation of template language for the radiologists to describe various common findings was also standardized which improved the efficiency and consistency among the radiologists and limited the referrals to those who may need clinical care. These changes in the review system emphasized whether or not there was a need for a clinical follow‐up on a finding, rather than on the findings themselves. Separating the doctor referral recommendation from the radiologist's summary allowed the radiologist to focus on their review, allowing the decision regarding the referral to be determined by local standards of practice. For example, the local practice (supported by medical literature) is that incidentally identified simple pineal cysts under 1 cm in size do not require follow‐up, therefore when found on a research scan, the recommendation is “you do not need to see your doctor about this report.” Conversely, more than a few white matter lesions could be related to hypertension or other more serious disorders. Therefore, when this finding is identified on a research scan, the recommendation is “please see your doctor about this report.” Thus, follow‐up recommendations are associated with potential diagnoses (appendix 3) following local standard of care. Uncommon findings are handled on a case‐by‐case basis. Importantly, both the radiology review and the cover letter stress that regardless of the referral recommendation, if a participant has any symptoms or medical problems, he/she should follow‐up with a primary care provider.

### Financial analysis

To quantify the financial burden of the radiology review process, an analysis was completed to calculate the cost from 2011 to 2014, as well as for the lifetime of the current process. The analysis included average weekly labor for all personnel (Medical Director, research operations, neuroinformatics engineer, information technology, and administrative staff), contracted radiologists, equipment, and the mailing supplies required to review scans and disseminate the results. The initial cost to develop a multisite imaging participant‐tracking system (COINS) was supported by external funding. The radiology review feature of the system was added at a later date to meet the IRB mandate. Ongoing support from the neuroinformatics and IT staff are limited to minor feature enhancements and troubleshooting. The vast majority of the expense (radiologist labor) for the radiology review process is built into the MRI service centers and included in the hourly scanning rate. The remaining expenses (IT, Medical Director, research operations, administrative staff, mailing costs, etc.) are covered through institutional indirect labor departments. Studies for which a more extensive review is requested or those that include nonbrain scans are billed for a portion of the additional review cost.

### Impact studies

#### Stakeholder perceptions

We conducted a retrospective survey of the perceptions and preferences of 396 investigators, research participants and IRB members (for full Methods, see Phillips et al. 2015). The survey design was based on previous empirical data related to IF disclosure and utilized both qualitative and quantitative assessments (Kirschen et al. 2006). The subjects included 196 former research participants who had received a single MRI report within the past 3–18 months, were not incarcerated, and were at least 18 years of age; 50 current and former IRB members from our institution's five external IRBs; and 100 current and former investigators, including research staff and external neuroimaging investigators.

#### Doctor referral outcomes study

We conducted a telephone survey of 40 participants who had received a radiology review within the past 2 years that included a finding for which clinical follow‐up was recommended. Adult, nonincarcerated participants were contacted via telephone to complete a brief survey of 12 semistructured questions. The survey content focused on the participant's experiences and the outcome of receiving his or her radiology review recommendation.

#### Data analysis

Summary statistics of single group continuous variables are presented as the averages (standard deviation), and categorical variables as percent comparisons. Comparisons of continuous variables across the stakeholder groups were performed using one‐way analysis of variance. The qualitative analyses were completed through group consensus coding to identify significant themes and subthemes prevalent in the data. The qualitative comments most representative of these themes are included within the text.

## Results

### Financial

Analyzed over the lifetime of the current process, the average total cost per MRI review has been approximately $23, with 70% of that cost being for radiologist labor. Because the radiologist reviews only the structural sequences performed as part of the research study (generally a high‐resolution T1, T2 or FLAIR sequence), the review time per scan is much less than for a clinical study and averages under 5 min per scan. From 2009 to 2011, the review cost per scan was ~$25 (Shoemaker et al. 2011). With further automation, the cost per review from 2011 to 2014 has dropped to ~$21. Although the cost per scan has decreased, the radiologist time per review has actually increased due to reviews of more comprehensive brain scans and the addition of nonbrain scans including those of the abdomen and lung, which typically include more images and higher finding rates (Gur et al. 2013; Klitzman et al. 2013; Ross et al. 2013; Lumbreras et al. 2014).

**Figure 1:**
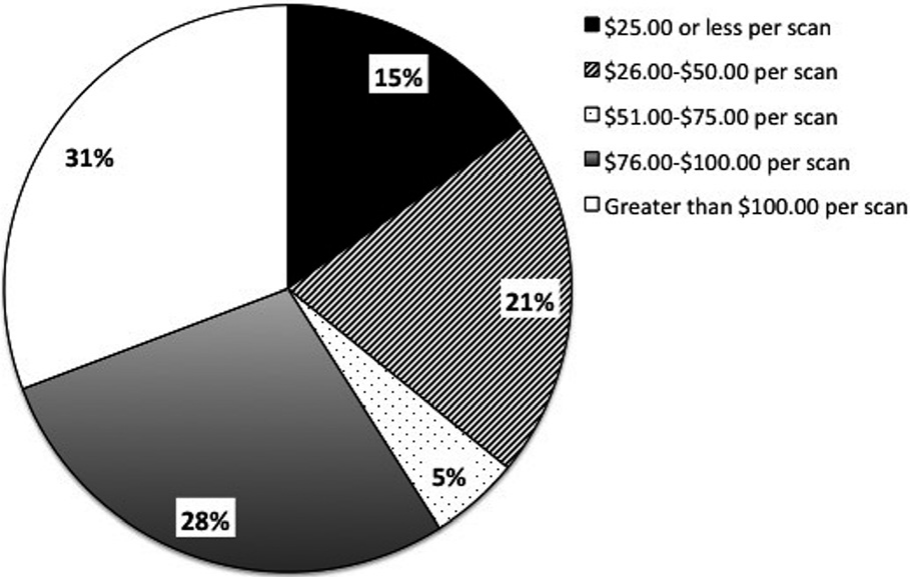
Investigator report of maximum acceptable cost per radiology review.

Our stakeholder perception study evaluated the opinions of IRB members and investigators regarding reasonable costs per scan if the IRB mandated radiology review of all research MRI scans (which is a current local mandate under which our organization operates). Reasonable cost was categorized into five options: $25 or less, $25–$50, $51–$75, $76–$100, and greater than $100 per scan. The results showed that the relevant stakeholders are largely unaware of the cost of the current system as indicated by the fact that 59% of investigators and 68% IRB members reported that they did not know or preferred not to answer. Of those participants who selected an amount, the responses were sharply divided: 32% of investigators and 13% of IRB members felt a cost greater than $100 per scan was reasonable. In contrast, 34% of investigators and 25% of IRB members felt a reasonable cost was $25 or less per scan. In addition, the investigators were asked an open‐ended question on the maximum allowable cost for reading and reporting scans. Their responses ranged from $0 to 1500/scan (m = 179.6, SD = 278.8). The distribution for maximum allowable cost was categorized into the five multiple‐choice options listed above and is presented in Figure 1.

This range in investigator's opinions regarding costs was further characterized using the qualitative data from our survey. All participants were given the opportunity to comment on their response regarding the reasonable and maximum allowable costs for radiology review. Two dichotomous themes emerged in these data. Some investigators expressed the opinion that they did not think cost should limit disclosure. A common statement from this group:

I do not think financial burden should be a deciding factor in deciding if we tell the participant.

Other investigators reported opposing sentiments as represented by:

Researchers should not have to use existing funding for [IF] screening.

Similarly, some investigators expressed resistance to performing mandated reads on scans on the basis of the potentially severe cost burden to studies. The following exemplifies this concern:

If IRB requires it, then IRB or the granting agency should be prepared to provide the funding for it.

### Health literacy and anxiety concerns

Several questions on the retrospective survey targeted the health literacy (HL) of the research participants, as well as the HL and health anxiety concerns from other relevant stakeholders in response to the participants receiving radiology review information. The estimates by the investigators, IRB members, and research participants differed significantly with respect to their perceptions of the HL of the research participants (*P* < 0.0001) and their ability to understand the research consent forms (*P* < 0.0001), the radiology cover letters, and the MRI reports (*P* < 0.0001). The participants consistently reported greater confidence in their own ability to understand these documents.

**Figure 2:**
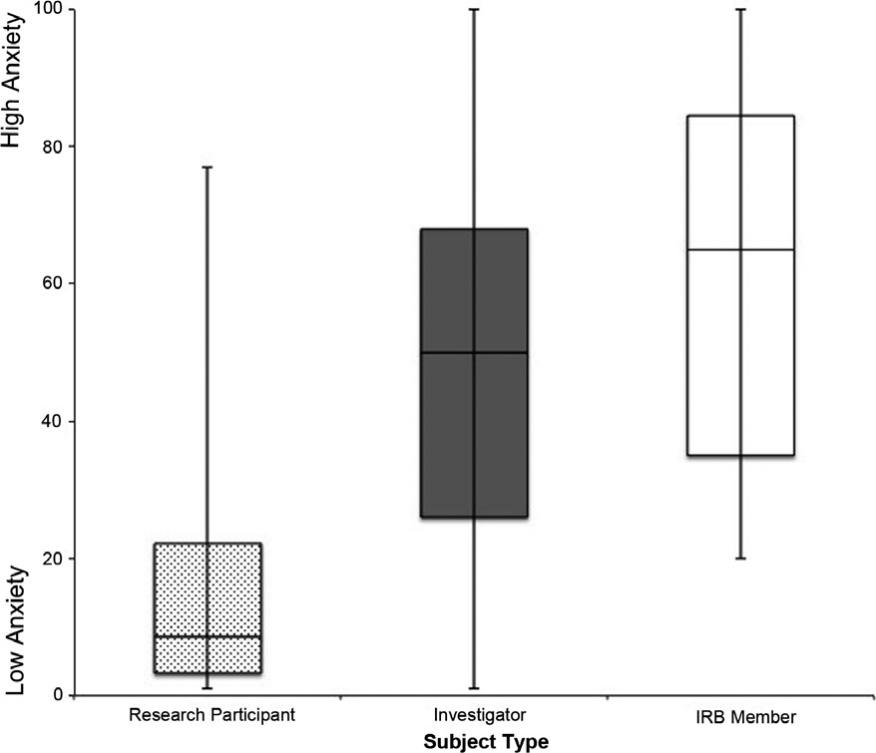
Research participant magnetic resonance imaging report anxiety.

Previously reported measures of participant MRI Report Anxiety Scores in response to receiving their MRI report resulted in low average anxiety scores with a mean = 15.2 (SD = 16.6) on a scale from 0 to 100, with 0 being very low anxiety. This was significantly lower than the estimates provided by both the investigators and the IRB members regarding the degree of anxiety participants would feel after receiving their MRI report (*P* < 0.001) (see Fig. 2).

Although the participants indicated a high level of understanding of the radiology letters and low measured anxiety, certain qualitative comments from the research participants' retrospective surveys suggest otherwise. The participants were given the opportunity to provide additional comments on their survey responses. A common theme within their responses was the shared sentiment that the radiology report letter was not accessible to the lay person. One participant stated:

The MRI scan results were difficult to understand because they were written in medical jargon. You should have a physician explain the results in plain language so they can be beneficial to the participants.

The research participants were not alone in this sentiment. Common themes from both the IRB member and investigators reflect similar concerns regarding the readability of the research documents (i.e., consent forms, MRI report). Both IRB members and investigators expressed concern that participants would not be able to understand their reports. A representative statement from an IRB Member:

The research participant receiving incidental findings alone would be unable to know the significance or even understand the meaning of the findings.

Investigators expressed similar concerns for participants' ability to comprehend their radiology reviews. One investigator commented:

I think that radiology and MRI reports would be completely foreign to most research participants so I would be concerned about their ability to read and completely understand that information on their own.

### Participants' desires for their findings

The research participants consistently expressed a desire to receive their MRI findings. Our retrospective survey reported that 87% of research participants expressed a preference to receive all scan findings, regardless of clinical significance, and 91% described it as a benefit to study participation (Phillips et al. 2015). When given the opportunity to comment on the potential benefit of reporting IF, all of our stakeholders, research participants, IRB members, and investigators, reported similar statements on the ethics involved in returning IF. In particular, a common theme among research participants was that they not only perceived the results as a benefit to research participation, but believed there was an ethical requirement to report personal health information. A few representative statements from research participants:

I do feel strongly that participants in the study who wish to know their medically important results should have the right to have those results revealed to them as fully as possible.

…the added benefit of getting medical advice –which I had a large cyst on my thyroid and luckily it turned out to be benign– but I would have never known to have it checked out otherwise, so I was glad to receive that and would be happy to participate in any other future studies.

Investigators reported similar themes in their comments with regard to the ethical requirement to disclose findings. A common theme among the investigator responses is demonstrated by the statement:

It is a benefit to them for their participation and an ethical need on the part of the research team.

IRB members reported similar statements regarding the potential for expanding participant autonomy by providing them with this information and that withholding this information would be ethically wrong. One IRB member expressed this shared sentiment:

I believe this is part of the protection of the human subject. If the researcher discovers something that may be of personal benefit to the participant, the researcher is obligated to provide that information to the participant.

### Health care impact of universal review and disclosure

In 2011, a comprehensive analysis of IF rates showed that that the rates at our institution were consistent with the extensive amount of publications on brain findings (Katzman et al. 1999; Vernooij et al. 2007; Royal and Peterson 2008; Illes and Borgelt 2009). From 2004 to 2011, the IF rate was 34% with the vast majority being for routine referrals and only 2.5% for urgent findings (Shoemaker et al. 2011). For the review period between 5/2013 and 12/2014 (*n* = 2251) under the new binary referral system, the doctor referral rate was 14.1%, despite increased numbers of scans of the abdomen and other areas with higher IF rates, as well as increased numbers of studies that included populations with higher known findings rates (e.g., Traumatic Brain Injury, Multiple Sclerosis). The doctor referral rates for the brain scans alone were 12.6%.

The retrospectively surveyed research participants received radiology reviews with recommendations of No Doctor Referral Necessary (*n* = 156, 80%) and Doctor Referral (*n* = 40, 20%). Of our total sample, 17 participants sought additional follow‐up care, although only 10 of these 17 had received a Doctor Referral recommendation.

Our Doctor Referral Outcomes Study involved a follow‐up phone interview with a second independent group of participants who had received a recommendation for a doctor referral. Of 40 participants interviewed, 38 remembered their radiology report recommending follow‐up care; however, only 63% of these participants followed the recommendation to see a physician.

The 37% of participants who did not seek follow‐up care listed various reasons for their decision: clinical reasons (14% – already knew about the finding or had been seeing a doctor for another health problem), financial reasons (29% – were waiting to get insurance or did not want to pay for doctor's visit), or a personal decision that the finding was not really significant (14%). However, the most common reason (43%) was that participants felt reassured after discussing their report with the Medical Director at the research institution. One of the participant's comments that summarizes this common sentiment:

I spoke with the neurologist, the doctor who called me afterwards… and he said it was not normal, but also not uncommon and most likely nothing. So he was saying he recommended that I see a doctor, but didn't say it was serious…

Among the 24 participants who followed up with their physician, nine participants (38%) received additional medical testing ranging from a second clinical MRI scan (*n* = 4), ultrasound (*n* = 2), CT scan (*n* = 2), and a fine‐needle aspiration (*n* = 1). While most of these participants incurred additional costs, none stated they regretted receiving their research MRI findings or pursuing follow‐up care. At the time of the interview, only two participants questioned whether the additional cost was worthwhile, but this reflected dissatisfaction with their current health care plan more than with their experience of receiving the research MRI report. The majority reported that even though the additional tests resulted in a financial cost, they were still glad to have received the report. A representative statement of this common theme:

For the peace of mind, it was absolutely worthwhile.

For a summary of results, see Table 1.

**Table 1:** Results summary

Financial	Internal researcher review cost $21/scan
	70% of cost supports radiologist labor
	IRB members/researchers lack consensus re: reasonable costs Range $0 to $1500 85% of researchers suggest reasonable cost is >$25
Health literacy	Discrepant assumptions (*P* < 0.0001) IRB members/researchers estimate that participants have *low* literacy Research participants self‐estimate *high* literacy
Anxiety	Discrepant assumptions (*P* < 0.001) IRB members/researchers estimate that research participants experience *high anxiety* from receiving a personal MRI report Research participants report *low anxiety* when actually receiving their own research radiology report
Return of results	87% of research participants want to be informed about all scan findings, regardless of clinical significance
	91% of research participants consider having their scans reviewed and findings reported to them a benefit to study participation
Incidental findings rates	14% of all scans recommended further clinical evaluation Binary categorization of IF into those needing or not needing further evaluation
	In outcomes study, 63% of participants followed up with a doctor as recommended in their MRI report
	37% of participants did not follow‐up as recommended 14% already knew of the IF 29% cited financial reasons for not seeing a doctor 43% decided not to follow‐up after discussing IF with the institution's Medical Director
Clinical follow‐up	38% of participants who followed‐up with a physician after receiving an IF report required further diagnostic studies None regretted receiving their IF report 2 were concerned re: high personal cost of additional testing

IRB, Institutional Review Board; IF, Incidental findings; MRI, Magnetic resonance imaging.

## Discussion

### Stakeholder perceptions

Our multimethod study confirmed existing empirical data regarding research participants' preferences for receiving information on IF and provided new insights into the broader stakeholder impact of the disclosure process. Research entities attempt to balance the importance of the autonomy of their participants (by providing them access to pertinent health information) with the realization that this information may be difficult to comprehend. It is clear that the participants desire this information and believe it is a benefit of their involvement in research; however, investigators and policy makers are left to question whether the participants really understand what to do with the radiology review information and know how to navigate the medical system when they need to access appropriate follow‐up clinical care.

The disparities between research participants, investigators, and IRB members regarding the readability of study documents and participants' HL suggests that further research is necessary to make objective determinations. As previously discussed, our measure of research participants' self‐estimate of their HL may not be an accurate description of participants' abilities (Phillips et al. 2015). The instrument utilizes questions such as “How confident are you that you can understand the directions on a bottle of Tylenol?” (Chew et al. 2004). The participants' self‐reported high level of confidence in this context may not reflect their actual ability to make appropriate healthcare decisions, specifically in the realm of neuroimaging findings.

This potential discrepancy is further highlighted by the research participant's qualitative comments describing their experience with their radiology report. These comments, echoed by both IRB members and investigators, suggest that the medical language of the radiology report is confusing to the participants. The challenge for imaging institutions and clinical care providers is that simplification of a radiologist's summary to reduce potential health anxiety and meet the health literacy demand of their participants may reduce the clinical utility of the radiology report. The radiologists who review the research scans need to provide full descriptions of any findings using accurate medical terminology to ensure that current and future medical professionals can provide the subject with appropriate follow‐up care. Therefore, institutions should not simplify the radiology review terminology, but instead should consider including an additional layman's summary or recommendation telling participants what to do with the information. Simply receiving a report stating “bilateral maxillary sinus opacification” or “small area of white matter hyperintensity” could be useful to a medical professional, but may confuse participants and potentially cause them unnecessary fear and anxiety. With the additional clarification and recommendation, “you do not need to see your doctor about this report” participants are given the medical information they desire, and are also given a suggestion on whether they should pursue follow‐up clinical care. The successful minimization of participant anxiety is demonstrated by the reportedly low levels of MRI report anxiety and the high numbers of participants who felt that the radiology report was a benefit to study participation. Although the participants do not always understand the radiologist's summary of findings, participants stated that they are not afraid to receive the report as a result of the clear recommendation and cover letter information. We believe the change in review process to the binary recommendation system to clarify the doctor referrals is partially responsible for mitigating these challenges.

### Financial and accessibility factors

It is now known that even low‐resolution MRI scans can identify IF, and as technology continues to improve, the chance of identifying IF greatly increases. National guidelines from the Bioethics Committee and NIH working groups suggest it is no longer acceptable not to plan for identification and disclosure of IF in neuroimaging studies (Illes et al. 2006; Underwood 2012). There is an absolute financial cost associated with review and disclosure, for which someone must pay. Imaging institutions should consider collaborating with the investigators to develop procedures to manage and budget the costs of the review and disclosure of IF. Our survey data demonstrate that the investigators and IRB members have little concept of what constitutes an appropriate cost, with the majority of both stakeholders responding that they did not have an estimate for this figure. For the local IRB requirements, it is interesting to note that although there is a mandate to review scans, the surveyed committee members were largely unaware of the financial cost to the study/institution to comply with the mandate. Among the investigators who responded to the question regarding the maximum allowable cost per scan to the research institution for reviewing and reporting findings, almost 85% responded with figures greater than the average cost at our imaging institution. Up to 30% of our investigators felt that costs greater than $100 per scan, approximately four times the current rate, would be allowable. These figures show that greater communication and education regarding the radiology review cost is needed for investigators and IRB members to understand how to determine appropriate budgets for IF management.

In addition to the financial discourse, investigators need to develop a plan to allow the radiologists and other specialists to complete the appropriate reviews and provide clinical support. Given the current remote access capabilities with which radiologists can review scans and physicians can interpret and provide consultation on the findings, imaging centers no longer need to be fully staffed with medical personnel or be directly associated with medical centers to provide these review services. In recent years, we have contracted with a collaborating nonmedical university imaging center to provide remote services for IF review as mandated by their IRB. If their MRI technologist flags a scan as being of potential concern, our institution provides priority reads on demand. The system allows the contracting institution to have customized templates for their cover letter and radiology report. Software is readily available to facilitate the scan transfer, radiology review, and disclosure process, and specialized hardware is no longer needed for this process. Administrative staff provides the majority of the ongoing labor for all sites, which is a small cost to the institution. For the small number of individuals who need additional clinical care, the e‐notifications and automated systems have streamlined our process and expedited the transition from participant to patient.

### Translation to clinical care

Research MRIs have known limitations when compared to clinical MRIs. The radiologists who evaluate research MRIs often must do so without access to additional health information, such as clinical exams, which would normally be provided in a clinical setting, and in many cases, even the structural scans may be less than fully comprehensive. Nevertheless, findings that may have clinical implications are never dismissed. Our study results reveal there are many motivations to participate in research; however, fewer than 5% of our survey participants indicated that they participated in an MRI research study because they were worried about a health condition. Based on our research populations, the health condition could have been the focus of the study in which they were enrolled. In rare cases, participants reported that they were concerned about a family/genetic condition (e.g., a sister was recently diagnosed with a brain tumor). When the MRI results are clear, the research findings should be disclosed and followed up accordingly. Discriminating between the findings that require additional medical testing and/or follow‐up and those that do not need additional attention is important. In collaboration with local specialists, the physicians who would receive such referrals are the clinicians who set the standards that determine the cases which need follow‐up.

Due to the large volume of scans at our institute, in particular those involving special populations, we have a unique opportunity to help redefine “normal” for MRI brain scans. The partnership with local specialty physicians to determine which findings need follow‐up care and which do not has allowed us to reduce unnecessary doctor referrals and participant burden. There is a perception that disclosing research MRI IF will overburden the health care system and result in additional participant risks and costs. In our retrospective survey, as well as in our outcomes phone interviews, participants considered the benefit to receiving IF information greater than the potential risk for additional costs associated with clinical follow‐up. Almost 92% of research participants not only consider the review not to be harmful, but instead consider it to be a benefit of study participation.

The results of our outcomes survey of former research participants regarding doctor referral rates were mixed – many participants did not seek additional care for their findings even when this was recommended in their radiology review report, and conversely, there were a number of participants who did follow‐up with a primary care physician despite a recommendation that it was not necessary. Overall, 8.7% of research participants (out of 196) decided to see a physician after receiving their radiology report, which represents the first empirical data available regarding impact on the local healthcare system of universal disclosure of research MRI results. Future work will focus on improving the appropriateness of follow‐up that occurs as a result of receiving a radiology report, possibly through providing targeted educational material with the report or, when the Medical Director is contacted, working harder to ensure that information is understood.

The information provided by the stakeholder groups provided valuable insight into a universal review and disclosure model and has also given our organization an opportunity to further revise and improve our IF management system and participant communication materials. The participants' specific constructive feedback on the radiology report letter content and format instigated immediate changes. Furthermore, the views of the investigators regarding the return of results to their participants have changed in a positive manner. There was significant resistance and concern for their participants in 2009 when the decision was made to return all results to participants (previously only doctor referral recommendations were returned) (Shoemaker et al. 2011). Now, our investigators are much more supportive of the process. This expanded study data demonstrate that a model of full disclosure is not only possible at nonclinical imaging institutes, but it does not cause significant amounts of harm (as reported by actual participants) and can be adapted to meet the needs of other areas of research as well as other organizations.

### Future directions for IF review and disclosure

The primary goal for our institution's IF review and disclosure process is to provide information to the participants in a manner that is meaningful and helpful, and without causing excess anxiety or burden. In a word, “beneficence.” This goal is accomplished through a system that is highly automated and requires minimal time or investment of resources. Using direct to patient medical records access (*Patient Portals)* as a leading example, we anticipate that our disclosure process will become more technology driven, with reports being provided to the participants through secure electronic means including links to additional resources tailored to their particular MRI findings. It is essential to develop supplemental educational materials about IF that are presented in a manner that conveys the complicated information in a way that individuals with all levels of health literacy can understand and use to make informed medical decisions.

## Conflict of Interest

None declared.

## Appendix 1 Example of radiology review cover letter and MRI report

### 1.1

The Mind Research Network

1101 Yale Blvd. NE

Albuquerque, New Mexico 87106

(505) 272‐5028

Date

Dear First Name Last Name,

Thank you very much for participating in imaging research! Research cannot occur without volunteers like you, and we very much appreciate your time and willingness to help.

The radiology report of your recent MRI scan is enclosed. It is written in medical language, so it might be confusing to you. As you review this report, please remember the following three points:

1. The MRI scan you had done was a research scan, which is different and in some ways not as clear as a medical scan that a doctor might order. So, things can be missed.
2. The research scan can sometimes show minor differences in how your body looks which may or may not be important, but are nonetheless present. Keep in mind that just as we all look different on the “outside,” we often look different on the “inside.”
3. Finally, and most importantly, no matter what the report states, please see your doctor if you have any symptoms or any health problems.

At the bottom of your enclosed radiology report is a recommendation about whether you should see a doctor about anything that was seen on your MRI scan. Please call me at the Mind Research Network (Phone Number) if you or your doctor have any questions.

Sincerely,

, MD

Medical Director

Board Certified in Neurology and Pediatrics

**Radiology Report**

Name: First Name Last Name

Age: XX

Sex: Female/Male

Scanner: Siemens TrioTim 3.0T

Scan Date: Date/Time

Study Name: IRB study Title

Study PI: PI First Name Last Name

**Radiologist's Summary:**

No Abnormal Findings

*This report was reviewed and electronically signed by Dr. (name), Board Certified in Radiology, on (date) at (time)*.

**Recommendation:**

You do not need to see your doctor about this report.

As always, please see your doctor if you have any symptoms or health problems. You may discuss this report with Dr., the Mind Research Network Medical Director, by calling (phone number). If he is not available, his assistant can tell you when to call back to speak with him.

*****This MRI scan was acquired for research purposes only and is not intended for diagnostic or clinical use *****

## Appendix 2 Sample consent form text regarding incidental findings

### 2.1

#### MRI Description section of consent

The MRI scan is being done to answer research questions, not to examine your brain for medical reasons. This MRI scan is not a substitute for a clinical scan (the type a doctor would order). The research scan might not show problems that may be picked up by a clinical MRI scan. However, all research MRI scans will be read by a neuroradiologist (a doctor with experience reading MRI scans) unless you have been scanned at MRN in the previous 6 months. When your scan is read, you will receive the official report by mail. If we find an abnormality that requires urgent follow‐up, we may contact you and your doctor (with your permission) by phone to help answer questions. Our Medical Director or the research team is always available to answer any questions you may have about your scan.

#### Costs for Participation section of consent

You will not be charged for any of the experimental study procedures, including the MRI scan. If incidental findings from the study result in the need for further evaluation/treatment, then you or your insurance company will be responsible for any additional clinical evaluation/treatment that may be needed.

#### Risks section of consent

Due to the high sensitivity of MRI in detecting abnormalities, there is a risk of false‐positive findings, identifying something on imaging studies that may or may not be important. This may result in anxiety and additional testing, possibly including a recommendation for clinical brain scans at your cost. The radiology report or other study data will not be put into your medical record unless you provide it to your physician. If the radiology report becomes part of your personal medical record, it may or may not have an effect on getting health insurance or life insurance in the future.

#### Benefits section of consent

In the course of this research, brain scans will be performed on you. These scans will be used solely for gathering scientific information for this study. During the study, you will not be provided a medical diagnosis or treatment for any brain condition, or other health problem. However, the radiologist may notice something in the brain tissue that could lead to early intervention if a problem were found.

## Appendix 3 Doctor Referral Recommendation List by Common Findings and Workflow

### 3.1

#### A You do NOT have to see your doctor about this report

Ear Nose Throat

- Mucus retention cyst
- Sinusitis
- Mucosal thickening of sinuses
- Scattered lymph nodes
- Prominent adenoids
- Deviated nasal septum
- Fluid in the sinuses

Brain

- Developmental venous anomaly
- Mild – moderate ventricular asymmetry
- Pineal cyst under 1 cm
- Cavum septum pellicudum
- Small to medium temporal lobe arachnoid cyst
- Small to medium posterior fossa cyst
- Low lying cerebellar tonsils less than 5 mm
- Areas of abnormal white matter signal
- Mildly prominent extra‐axial fluid spaces
- Cerebral volume less than expected for age
- Abnormal pituitary signal (hypointense, hyperintense, heterogeneous)
- Pituitary cyst
- Pituitary prominence
- Concave margin of pituitary gland

#### B Please see your doctor about this report

Ear Nose Throat

- Complete opacification of one sinus
- Unusual appearance of lymph node, greater than 1 cm in size

Brain

- Any structural lesion (r/o tumor)
- Arteriorvenous Malformation, aneurysm, cavernous malformation
- Pituitary enlargement
- Chiari malformation (tonsils extending below 5 mm, or if less than 5 mm but the brainstem looks “tight”)
- Large brain cyst
- Significant white matter signal change

#### C Workflow

- Clear finding: if at all possible match the finding with one of the diagnoses above – triggers automatic recommendation
- Unclear finding: suggested recommendation with report sent to the Medical Director to make final decision, may require outside specialist discussion.
- Medical Director reviews all “please see your doctor” reports as well as unclear findings; over time, the template readings may be revised
- Participants may reach the Medical Director by calling MRN front desk, if not available, they will be given a time to call back
